# Epidemiology and genetic diversity of *Streptococcus suis* in smallhold swine farms in the Philippines

**DOI:** 10.1038/s41598-023-48406-9

**Published:** 2023-12-01

**Authors:** Susan A. Sedano, Mary Grace Concepcion T. Cantalejo, Christine Grace Angela R. Lapitan, Angelo Miguel Elijah S. de Guzman, Jennielyn T. Consignado, Nancy A. Tandang, Maria Amelita C. Estacio, Anusak Kerdsin, Benji Brayan Ilagan Silva

**Affiliations:** 1 Veterinary Vaccines Laboratory, National Institute of Molecular Biology and Biotechnology (BIOTECH), University of the Philippines Los Baños, 4031 Los Baños, Laguna Philippines; 2 Ecosystem Services and Environmental Policy Laboratory, School of Environmental Science and Management, University of the Philippines Los Baños, 4031 Los Baños, Laguna Philippines; 3 Department of Agrarian Reform, Elliptical Road, Diliman, 1107 Quezon City, Philippines; 4 Institute of Statistics, College of Arts and Sciences, University of the Philippines Los Baños, 4031 Los Baños, Laguna Philippines; 5 Department of Basic Veterinary Sciences, College of Veterinary Medicine, University of the Philippines Los Baños, 4031 Los Baños, Laguna Philippines; 6 Faculty of Public Health, Kasetsart University, Chalermphrakiat Sakon Nakhon Province Campus, Sakon Nakhon, 47000 Thailand

**Keywords:** Microbiology, Zoology

## Abstract

This study aimed to determine the presence and characteristics of locally circulating strains of *Streptococcus suis*, the most important streptococcal pathogen in swine. Oral swab samples were collected from pigs from 664 representative smallhold farms across nine provinces in the Philippines. Isolates were identified and characterized using PCR assays. The study revealed an isolation rate of 15.8% (105/664, 95% CI: 13.0–18.6) among the sampled farms. Two hundred sixty-nine (269) *S. suis* isolates were recovered from 119 unique samples. Serotype 31 was the most prevalent (50/269, 95% CI: 13.9–23.2) among the other serotypes identified: 5, 6, 8, 9, 10, 11, 15, 16, 17, 21, 27, 28, and 29. The detection of the three ‘classical’ *S. suis* virulence-associated genes showed that 90.7% (244/269, 95% CI: 87.2–94.2) were *mrp*^*-*^*/epf*^*-*^*/sly*^*-.*^ Multilocus sequence typing (MLST) analysis further revealed 70 novel sequence types (STs). Notably, several local isolates belonging to these novel STs formed clonal complexes (CC) with *S. suis* strains recovered from Spain and USA, which are major pork-exporting countries to the Philippines. This study functionally marks the national baseline knowledge of *S. suis* in Philippines.

*Streptococcus suis,* a Gram-positive bacterium, is the most important streptococcal pathogen affecting swine industries worldwide^1^. Pigs infected with virulent strains of this bacterium suffer from various clinical manifestations, including meningitis, arthritis, endocarditis, septicemia, pneumonia, and/or sudden death^2^. Despite the dense pig population in Southeast Asian countries and cultural practices involving the consumption of raw and undercooked pork in countries like Thailand, Vietnam and Laos, limited data on *S. suis* infections in pigs are available for countries such as Singapore, Philippines, Laos, and Cambodia^3–5^. This is of great concern as the number of human cases of *S. suis* infection has been increasing in this region^3,4^. Several major outbreaks of human infections were recorded in Thailand and China, resulting in deaths and significant economic loss to the affected provinces^6,7^.

In the Philippines, between 47 and 60% of the animal meat consumption of Filipinos comes from pork^8^. For years, the Philippines has been among the top pork producers worldwide, with an estimated value at around PHP 270 billion in 2021 and a pig population of around 10.18 million as of March 2023^8–10^. Smallhold farms consistently account for most of the pork production in the Philippines, contributing between 62 and 78% of the total swine inventory annually, spanning from 2000 to 2023^10^. However, this farm type is susceptible to significant losses due to limited practice of biosecurity measures such as vaccination, footbath provision, perimeter fencing, rodent control, and swill feeding management^11^.

In Huong et al*.*^12^, the Philippines, considering its wide and intensive hog raising practice, was identified as the Asian gap in information on *S. suis*. This is an important call that necessitates action particularly since multiple cases of human infections of *S. suis* have already been reportedly contracted in the country, including a number of cases diagnosed abroad^3,13–18^. This highlights years, if not decades, of gap in *S. suis* research data relevant to livestock health and disease monitoring, human health risks, and emergence and changes in antimicrobial resistance pattern in the country.

As a response, this study aimed to investigate the presence, distribution, and characteristics of *S. suis* in smallhold farms in the Philippines, specifically, the locally circulating serotypes, virulence gene profiles, and sequence types of the bacterium in the country.

## Results

### Isolation of *Streptococcus suis*

**Figure 1:**
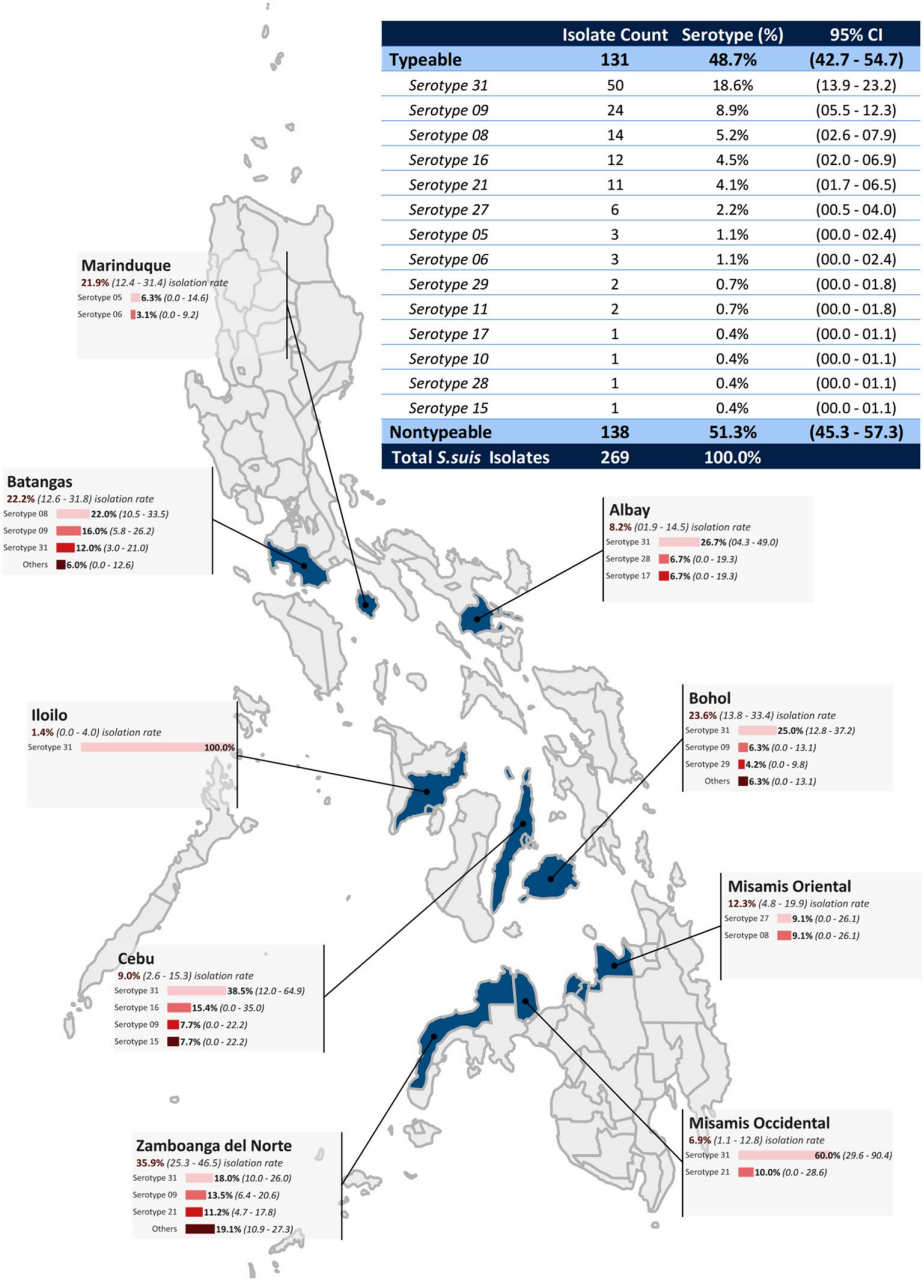
Geographical distribution of the recovered isolates of *Streptococcus suis* among nine provinces in the Philippines. The map was created using MapChart software (https://www.mapchart.net).

Out of the 664 farms surveyed, *S. suis* isolates were identified from 105 (15.8%, 95% CI: 13.0–18.6) farms using the *S. suis*-specific, *recN-*based PCR assay. None of the farms can be positively identified as affected by *S. suis* clinical infections. The observed proportion of farms with positive isolation of *S. suis* is 35.9% (28/78, 95% CI: 25.3–46.5) in Zamboanga del Norte, 23.6% (17/72, 95% CI: 13.8–33.4) in Bohol, 22.2% (16/72, 95% CI: 12.6–31.8) in Batangas, 21.9% (16/73, 95% CI: 12.4–31.4 ) in Marinduque, 12.3% (9/73, 95% CI: 4.8–19.9) in Misamis Oriental, 9.0% (7/78, 95% CI: 2.6–15.3) in Cebu, 8.2% (6/73, 95% CI: 1.9–14.5) in Albay, 6.9% (5/72, 95% CI: 1.1–12.8) in Misamis Occidental, and 1.4% (1/73, 95% CI: 0–4.0) in Iloilo. Zamboanga del Norte, which was estimated to have the highest positive isolation rate at provincial level, was significantly different from Misamis Oriental, Cebu, Albay, Misamis Occidental, and Iloilo. However, it was not significantly different from Bohol, Batangas, and Marinduque, as reflected by the overlapping interval estimates among these provinces. Alternatively, Iloilo, which posted the lowest positive isolation rate, was significantly different from Misamis Oriental, Marinduque, Batangas, Bohol, and Zamboanga del Norte but was not statistically different from Cebu, Albay, and Misamis Occidental (Fig. 1, Supplemental Fig. 1). Among the 105 *S. suis* positive farms, the proportion of sampled pigs positive for *S. suis* ranged from 13 to 100% per farm.

Comparing the proportions of *S. suis-*positive pigs by age group, isolation from piglets and sows outnumber isolation proportions from growers and boars. Results showed that 10.4% of piglets (45/433, 95% CI: 7.5–13.3), 8.1% of sows (36/447, 95% CI: 5.5–10.6), 5.6% of growers/finishers (37/660, 95% CI: 3.9–7.4), and 3.7% of boars (1/27, 95% CI: 0.0–10.8) tested positive for *S. suis* by isolation. However, the observed difference in the estimates could not support the idea that any age group is statistically more likely to be *S. suis* positive, as shown by the overlapping interval estimates (Supplemental Fig. 2).

In total, swab samples were individually collected from the oral cavities of 1,567 pigs, particularly from the surface of the palatine tonsils. Among these samples, 269 *S. suis* isolates were recovered from 119 unique samples (7.6%, 95% CI: 6.3–8.9). All isolates were recovered from asymptomatic pigs, except for two isolates obtained from a deceased piglet that exhibited fever symptoms before coincidentally dying on the day of sampling. No additional information was available for these two isolates that would confirm clinical *S. suis* infection. Therefore, these two isolates were characterized together with all other isolated *S. suis* strains. No isolates recovered from systemic or invasive clinical infections of *S. suis* were included.

**Figure 2:**
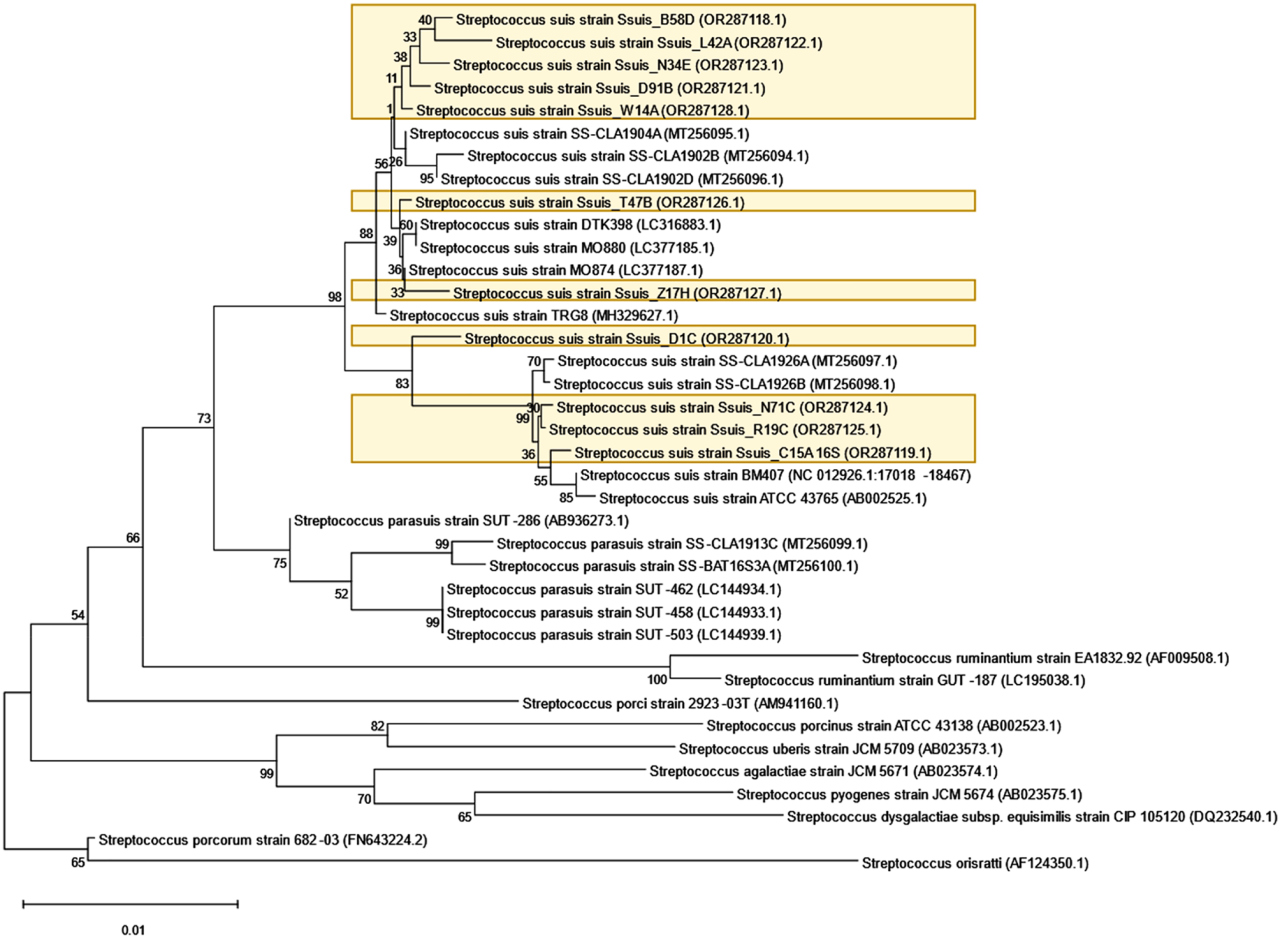
Phylogenetic relationship of recovered *Streptococcus suis* isolates and representative reference streptococcal strains, as inferred from the *16 s rRNA* gene sequence comparisons using the neighbor-joining method.

To further validate the identity of the *S. suis* isolates, 11 representative isolates were subjected to *16 s rRNA* gene sequencing. BLAST analysis revealed a 100% query cover and a 99 to 100% percentage identity (PIaD) to *S. suis*. All sequences were submitted to NCBI with GenBank accession numbers OR287118–OR287128. Figure 2 shows the phylogenetic tree constructed from the partial *16 s rRNA* gene sequences of the recovered isolates, alongside several type and representative sequences from other streptococcal species.

## Characterization of recovered *Streptococcus suis* isolates

### Molecular serotyping

From the 119 unique swab samples gathered from the sampled animals, 269 *S. suis* isolates were recovered. The identification of the capsular polysaccharide (*cps*) types of *S. suis* was carried out using a four-reaction multiplex PCR assay^19^.

Among the recovered isolates, 131 of isolates tested were assigned to the 29 recognized serotypes, while the remaining 138 isolates could not be classified into any specific serotype and were categorized as nontypeable isolates. Nontypeable isolates were the most prevalent, followed by serotype 31. The proportion of the nontypeable strains (138/269, 51.3%, 95% CI: 45.3–57.3) was significantly higher than any of the typeable strains, as evidenced by their 95% confidence intervals. Additionally, among typeable strains the proportion of serotype 31 strains (50/269, 18.6%, 95% CI: 13.9–23.2) was significantly higher than all other detected typeable serotypes. Furthermore, the proportion of serotype 9 strains was significantly different from serotypes 31, 27, 5, 6, 29, 11, 17, 10, 28, and 15 but not significantly different from serotypes 21, 16, and 8. Subsequently, the proportion of serotype 27 strains was significantly different from serotypes 9 and 31 but not significantly different from the rest of the typeable strains (Supplemental Fig. 3).

**Figure 3:**
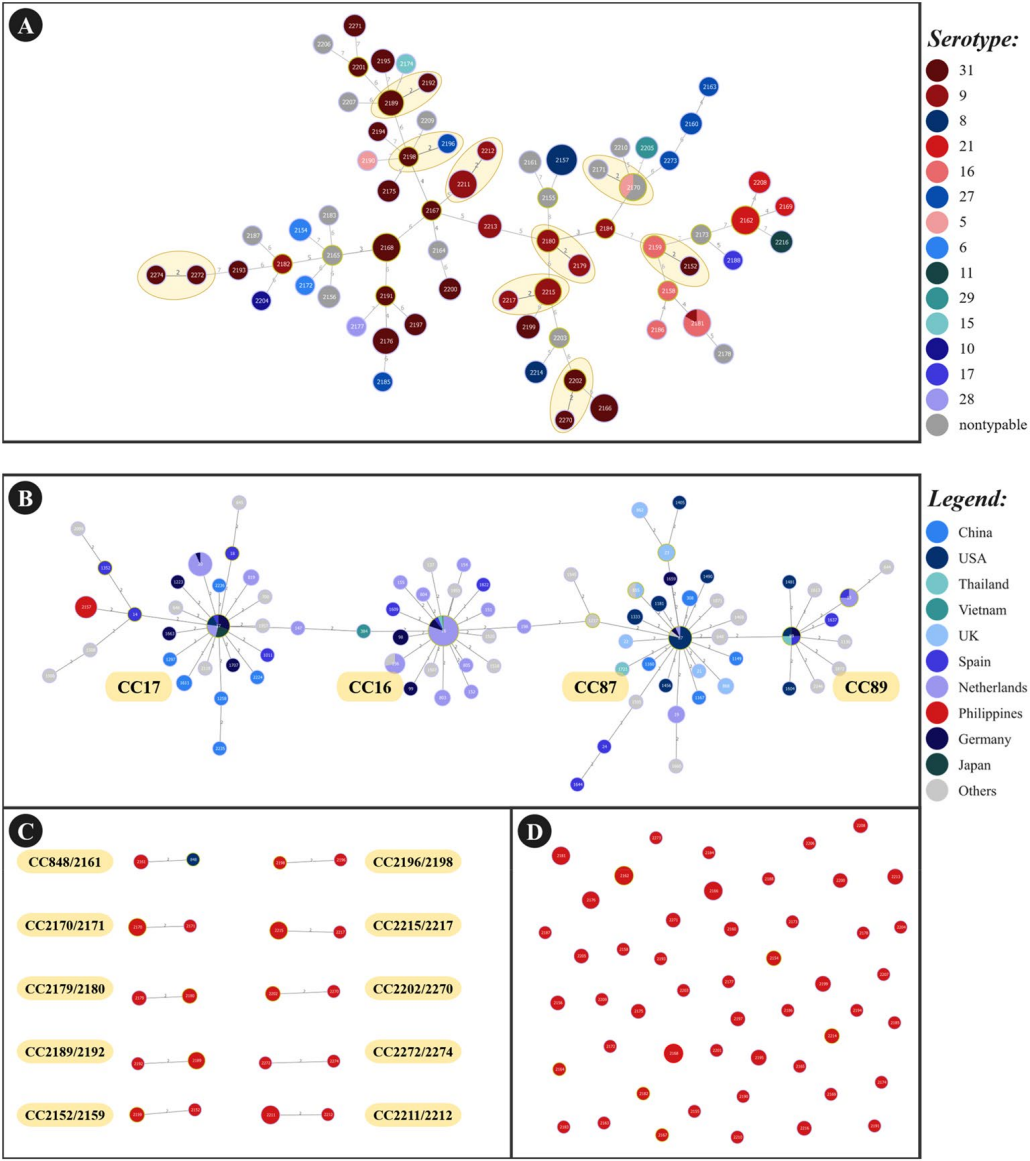
Population snapshot of *Streptococcus suis* in the Philippines showing (**A**) minimal spanning tree representing the serotype distribution of STs and the newly identified clonal complexes, (**B**) the relationship of a novel ST with previously recognized clonal complexes (**C**) the newly identified clonal complexes, and (**D**) singletons.

### Virulence gene profiling

Only 9.3% (95% CI: 5.8–12.8) of isolates carried at least one of the three classical virulence-associated genes (VAGs), namely *mrp, epf,* and *sly*. The predominant genotype detected is *mrp*^*-*^*/epf*^*-*^*/sly*^*-*^ (90.7%, 95% CI: 87.2–94.2). Other observed genotypes include *mrp*^*-*^*/epf*^*-*^*/sly*^+^ (n = 11) and *mrp*^*-*^*/epf*^+^*/sly*^*-*^ (n = 14). The *sly-*positive isolates were associated with serotypes 6, 11, and 15. Other *sly-*positive isolates were found among nontypeable strains. The virulence-associated gene *epf* was observed in serotype 8 (n = 11), serotype 27 (n = 2), and serotype 31 (n = 1). It could also be important to note that 52.0% (95% CI: 32.4–71.6) of isolates positive for VAGs were recovered from sows and piglets.

### Multilocus sequence typing

From the 269 *S. suis* isolates, 145 were selected for multilocus sequence typing (MLST) using seven different housekeeping genes (*aroA*, *cpn60*, *dpr*, *gki*, *mutS*, *recA*, and *thrA*). The sequences were submitted to pubMLST database, and analyses revealed 101 novel alleles. Specifically, 14 new alleles were found in the *aroA* gene, 19 in the *cpn60* gene, 10 in the *dpr* gene, 17 in the *gki* gene, 17 in the *mutS* gene, 13 in the *recA* gene, and 11 in the *thrA* gene.

Interestingly, it was determined that all the 145 isolates could not be typed into any of the previously reported STs available in the database and were subsequently assigned to 70 novel STs. The most common ST observed was ST2157, which was observed in 11 isolates, followed by ST2162, ST2166, and ST2211, each with seven isolates. Meanwhile, ST2168 and ST2181 have six isolates each, and ST2170, ST2176 and ST2215 were represented in the collection by five isolates each. Further analysis of the association between serotypes and STs (Fig. 3.A.) showed that eight out of the 14 identified serotypes exhibited multiple STs. Serotype 31, the predominant serotype, exhibited 22 different STs. These include ST2152, ST2166, ST2167, ST2168, ST2175, ST2176, ST2189, ST2191, ST2192, ST2193, ST2194, ST2195, ST2197, ST2198, ST2199, ST2200, ST2201, ST2202, ST2270, ST2271, ST2272, and ST2274. The number of distinct STs within the other serotypes range from one to 10 (Fig. 3.A).

It was also determined that ST2154, ST2174, ST2196, ST2172, and ST2157 isolates tested positive for at least one VAG. These five STs were specifically linked to certain serotypes: ST2174 with serotype 15; ST2196 with serotype 31; ST2172 and ST2154 with serotype 6; and ST2157 with serotype 8. A clonal complex (CC) refers to a group of two or more independent isolates sharing identical genetic alleles at five or more loci^20^. Eleven (11) distinct CCs among the 145 isolates were identified using PHYLOViZ software goeBURST algorithm and illustration^21^: CC2217/2215, CC2159/2152, CC2179/2180, CC2211/2212, CC2189/2192, CC2170/2171, CC2272/2274, CC2198/2196, CC2270/2202, CC848/CC2161, and CC17 (Fig. 3A–C). ST2157 is related to CC17 via ST14. CC16 is related to CC87, whereas CC87 is closely related to CC89 (Fig. 3B). The remaining 50 STs were observed to be singleton isolates, which were not associated with any CCs (Fig. 3D).

Notably, two STs from the Philippines were connected to two other STs, one from Spain and another from USA. Specifically, ST2157 from Philippines belongs to CC17 via ST14 from Spain, while ST2161 from the Philippines formed a clonal complex with ST848 from USA. A total of 7.6% isolates (11/145) belonged to CC17 and 1.4% isolates (2/145) belonged to CC848/CC2161.

## Discussion

Pigs and wild boars are considered natural reservoirs of *S. suis*, and colonization is generally regarded as present in almost all herds^1,22^. An individual pig, whether diseased or asymptomatic, may carry a mixture of multiple and diverse *S. suis* strains colonizing the upper respiratory tract, particularly the tonsils and nasal cavities, as well as the vaginal and possibly the alimentary tract^1^. The transition of *S. suis* from a colonizing pathobiont into an invasive pathogen is driven by a complex multifactorial process influenced by host health, environmental conditions, and strain-specific factors^22,23^.

In diseased pigs, examination of the clinical manifestation, the age of the affected individuals, and the characteristics of macroscopic lesions are common reference points for a presumptive diagnosis of *S. suis* clinical infections. This diagnosis is only confirmed when microscopic lesions typical of *S. suis* infection are observed, and isolation of *S. suis* is achieved from tissue samples, preferably from multiple organs or sites^1^. Meanwhile, in asymptomatic pigs, the recovery of *S. suis* strains from the nasal cavities or tonsils suggests a high load of this bacterium in these sites. This is because microbiological isolation is of low sensitivity for detection due to the presence of competing members of the microbiota. Isolation of *S. suis* strains from these sites may, therefore, indicate active transmission rather than a carrier state^1,24,25^. However, this distinction has not consistently been applied in the literature such that the proportion of pigs in a sampled population that is culturally positive for *S. suis* has also been referred to as the carriage rate, while the individual animals have also been called carrier pigs^26–29^.

It is crucial to note, however, that such colonizing isolates of *S. suis* from asymptomatic pigs should not be automatically considered avirulent or non-pathogenic strains. The classification of *S. suis* isolates as virulent or avirulent, pathogenic, opportunistic, or strictly commensal is a complex issue that has previously been problematized and discussed. In brief, there is no standard definition of what a virulent strain is. Asymptomatic pigs may carry virulent strains, particularly taking into account that most human cases of infections are acquired due to the consumption of contaminated pork products that are presumably from healthy pigs. Further complicating this problem is the absence of standardized assessment methods and models, either in vivo or in vitro, to assess strain virulence or evaluate the importance of suggested critical virulence factors, which would enable direct comparison of results^30^.

As such, for both clinical infections and asymptomatic colonization of *S. suis*, direct detection of *S. suis* by molecular detection from tonsils or nasal cavities offer limited practical utility^1,31^. On the other hand, while microbiologic isolation of *S. suis* strains may be less sensitive than molecular or serologic detection, isolation is a requirement for the confirmation of clinical infection and identification of potentially actively transmitted strains in asymptomatic pigs^1^. Furthermore, recovered isolates, regardless of the clinical condition of the source, offer opportunities for further genomic and phenotypic characterizations, including but not limited to serotyping, detection of virulence-associated genes, MLST, host challenge assays, and antimicrobial resistance, among others. These pieces of information are of utmost importance in the characterization of the epidemiology of *S. suis* and in the holistic evaluation of the importance of circulating *S. suis* strains*.* Microbiologic isolation of *S. suis* strains, therefore, has been utilized in numerous studies across various countries to describe the prevalence and characteristics of *S. suis* in both diseased and asymptomatic pigs^28,32–42^.

Almost a decade after Huong et al*.* (2014) singled out the Philippines as the *S. suis* knowledge gap in Asia, this study presents a representative cross-sectional survey in smallhold swine farms in the country^12^. No prior information on outbreaks, if they had occurred, or list of farms with a history of *S. suis*-related diseases was available to guide the selection of farms for sampling. Instead, smallhold farms were sampled from high pig population density provinces in the country.

The observed positive isolation rate among pigs in the current study (119/1567, 7.6%, 95% CI: 6.3–8.9) is comparable to an initial report of *S. suis* isolation in the Philippines in 2020, which achieved a 9.09% (4/44, 95% CI: 0.6–17.6) isolation rate from a single farm^8^. However, this rate is higher than an older report detailing zero recovered isolates from 220 nasal and tonsil swabs of slaughtered pigs from a province not included in the current study^43^. Similarly, reports on *S. suis* isolation from healthy pigs from farms or slaughterhouses in other countries, such as Northern Vietnam^44^, China (Jiangsu)^45^, Northern Thailand^46^, and Turkey^43^, showed isolation rates ranging from 0 to 6.0% . On the other hand, reports from China (Anhui)^47^, Thailand (Chiang Mai)^48^, and China (Xinjiang)^47^ ranged from 7.8 to 8.3%. Lastly, isolation rates reported from other provinces in China^47^, Korea^49,50^, India^51^, Spain^52^, Canada (Quebec)^53^, Thailand (Phayao)^27,54^, UK^26^, Northern and Central Thailand^28,46^, Southern Vietnam^36^, and Canada (Ontario)^55^ ranged from 11.2 to 73.1%, demonstrating a wide range of reported *S. suis* isolation rates in the literature (Supplemental Fig. 4).

Currently, 29 serotypes of *S. suis* are recognized based on the serological characteristics of the bacterial capsular polysaccharide^56–59^. Among these serotypes, serotype 2 is most associated with infections in both pigs and humans. It has been determined to be responsible for over 80% of human cases, putting emphasis on its importance as a global zoonotic threat^60^. As a result, a significant portion of the research and reports has primarily focused on *S. suis* serotype 2 samples obtained from diseased pigs and humans. Globally, the predominant *S. suis* serotypes in clinical pig cases are serotypes 2, 9, 3, 1/2, and 7. However, within North America, serotypes 2 and 3 were the predominant serotypes, followed by serotypes 1/2, 8, and 7^3,30,61^. This can be attributed to the fact that the distribution of serotypes exhibits significant variations over time and in different geographical areas^41^.

In this study, no serotype 2 strains were recovered. Serotype 31 (38.17%; 50/131) was detected in 7 out of 9 provinces sampled, which may suggest that there is a need to put serotype 31 under surveillance to further understand its clinal and epidemiological importance, and identify potential control measures, if necessary. This is particularly important since other studies in Vietnam, Thailand, Spain, Canada, UK, and China only reported isolation rates of serotype 31 strains ranging from 0 to 7.2% in healthy or asymptomatic pigs^28,26,62^. On the other hand, there have also been several reports of serotype 31 associated with diseased pigs in Canada^42^ and China^63^ (Supplemental Fig. 5a). Notably, this serotype has also been reported to have caused a human infection in Thailand^56^.

Furthermore, over the last two decades, there has been an observed increase in the prevalence of serotype 9 isolates from diseased pigs in Netherlands^33^, Spain^39^, and Canada^62,64^, and it is also among the most commonly isolated serotypes in both clinically healthy and diseased pigs in commercial farms in China^65^. Reported proportion of serotype 9 isolates from diseased pigs ranged from 0–47.3% as reported from studies in Canada^42,55,62^, Spain^39^, Brazil^66^, Taiwan^67^, and the Netherlands^68^. Meanwhile, studies on healthy or asymptomatic pigs in Canada^55,62^, China^26^, Vietnam^36^, Korea^49,50^, Thailand^27,28,54^, UK^26^, and Spain^52^ ranged from 0 to 15.9% (Supplemental Fig. 5b).

Meanwhile, serotype 8 is reported to be isolated in diseased pigs from Spain, Brazil, Canada, Taiwan and Korea^39,49,62,66,67,69^. Serotype 8 ranked 5th as the most predominant and pathogenic serotype in diseased pigs worldwide from 2002 to 2013^3^. Serotype 16, on the other hand, has also been reportedly isolated from diseased pigs in Germany and South Korea^70^. Lastly, serotype 21 is also recovered in clinically healthy pigs in North America but is associated with diseased pigs in Canada^42,71,72^. Variable detection rates of serotypes 8, 16, and 21 both from diseased and non-diseased samples have been reported in the literature^27,28,36,39,42,49,50,52–55,26,62,66–68^. Detection rates of serotype 8 strains ranged from 0 to 54.0%, while serotypes 16 and 21 were reportedly detected at 0–24.7% and 0–6.3%, respectively (Supplemental Fig. 5c–e).

The predominance of nontypeable strains in the current study aligns with findings from other publications reporting that nontypeable *S. suis* strains are commonly isolated in field conditions. These strains may be considered as potential novel serotypes or mutants of known serotypes^19,73,74^. Studies demonstrated that unencapsulated strains, which include nontypeable isolates, exhibit unique properties such as increased adherence to surfaces and cells, as well as the ability to form biofilms, which may contribute to their persistence and transmission^75^. Nontypeable isolates are also frequently recovered from both clinically ill and healthy pigs^74^. Although available information is limited, there have been reports of nontypeable *S. suis* isolates from pigs with meningitis in China (designated novel serotype variant Chz)^76^, and a nontypeable unencapsulated *S. suis* strain has also been reported in a human case in Thailand^77^. Hence, it is also important to consider potential risks associated with nontypeable isolates, considering that their role in disease transmission could not be disregarded.

The predominant *S. suis* VAG genotype (90.71%; 244/269) identified in this study is *mrp−/epf−/sly−.* This is consistent with other reports on *S. suis* isolated from healthy/carrier pigs from China^78^, Thailand^28^, and Germany^35^, forwarding the observation that isolates positive for *epf, mrp,* and *sly* genes were significantly less frequently detected in clinically healthy pigs^35^. It is important to note, however, that this determination does not automatically indicate avirulence, since most virulence-related studies that considered *epf, mrp,* and *sly* as the main VAGs primarily studied serotype 2 strains^79^. In fact, the *mrp*+*/epf*+*/sly*+ genotype is almost always reported in serotype 2 strains isolated from diseased pigs in Europe and Asia^34,80^. Moreover, it has been suggested that *mrp, epf,* and *sly* are coincidentally associated with virulence rather than being the actual determining factors of virulence, which means the absence of one or more of these proteins does not necessarily result in lack of virulence^30,81^. A more recent whole genome and pan-genome analyses suggested that *ofs* (encoding for serum opacity factor) and *srtF* (encoding for sortase F) are stronger predictors for differentiating pathogenicity compared to 71 other previously suggested VAGs in *S. suis*, including *mrp, epf,* and *sly*^82^.

Among the 145 *S. suis* isolates that were examined by MLST, no previously known STs were found. These isolates were submitted to pubMLST and were assigned as 70 novel STs. Generally, *S. suis* isolates from clinically healthy pigs are not subjected to MLST, which could partially account for the numerous novel STs in this study^83^. Of note, two specific STs were found to have connections to STs from other countries. ST2157 isolates, which were recovered from the dead piglet, was linked to ST14 from Spain, a serotype 3 strain isolated from a lung tissue showing pneumonia. The diseased piglet was not reported to exhibit manifestations of pneumonia but was only noted to have symptoms of fever before sudden death. On the other hand, ST2161 was connected to ST848 from the United States of America (USA), which was isolated from a pig in 2015. Considering these observed connections, it is tempting to hypothesize if importation and international trade could also be important factors to account for the diversity of *S. suis* in the Philippines, given that pork and pig products are also common sources of *S. suis* infections^84^. Based on the data obtained from the Foreign Agricultural Service (FAS) under the US Department of Agriculture (USDA), the Philippines has maintained a longstanding practice of importing live pigs from the United States, with a cumulative total of pigs imported at 14,689 heads from 1967 to 2022. The Philippine Statistics Authority (PSA) reported that a total of 2,703 live pigs were imported mainly from Canada, Spain, Netherlands, Brazil, and the USA 2018 to 2020. The problem, however, is that there is no information on the movement of these imported animals within the Philippines making it impossible to prove the connection of importation and the observed relationship among Philippine isolates sharing a clonal complex with isolates from other countries.

Pending the availability of information on the predominant serotypes of *S. suis* from clinical cases of infections, the current results, considering the observed predominant serotypes and the entirely novel set of MLST, suggest a potentially different epidemiology of *S. suis* in the Philippines^34,41,49,38^.

Smallhold farms contribute about 80% of the total hog production in the Philippines. Despite this, the scant information on important bacterial pathogens, especially those that pose zoonotic risks like *S. suis*, coupled with the limited implementation and understanding of biosecurity-related practices and measures, point to an increased danger of transmission of the pathogen among animals, and at the same time, from animals to humans.

Altogether, the current findings, which provided detailed molecular characterization of circulating *S. suis* strains in the Philippines for the first time, are highly relevant to both veterinary and human medicine, especially for individuals with direct contact with pigs and pork products, including pig farmers, abattoir workers, as well as the general consumers^3,12,28,35,61^. However, the current study is not without limitations, which must be given attention with further research studies. Particularly, subsequent studies may focus on clinical isolates of *S. suis*, including historical isolates (if present in regional or national diagnostic laboratories) and newer isolates recovered from more recent cases. Thus, this also emphasizes the importance of continuous surveillance of this pathogen among farms, and even among slaughterhouses and markets in the country, as performed in other countries before^35,36,60^. Also, in consideration to the growing number of studies indicating the propensity of *S. suis* for rapid development of multidrug resistance^27,85–95^, characterization of local isolates must also be extended to cover phenotypic and genotypic antimicrobial resistance characteristics to gain an understanding on the applicability and efficacy of currently implemented practices regarding antibiotic usage. Finally, whole genome sequencing and related bioinformatics studies on local isolates, particularly those that were identified as epidemiologically or clinically significant, would further bolster grounded local understanding of this important pathogen.

With plans to further intensify the swine raising industry in the Philippines, the current study provides a snapshot of the on-going conditions related to *S. suis* infection in key hog-raising provinces in the country. This study functionally marks the national baseline knowledge on *S. suis* in Philippines.

## Methods

### Ethics statement

Ethics approval for the research, specifically for the conduct of and the protocol for sample collection from farm animals, was obtained from the Institutional Animal Care and Use Committee (IACUC) of the University of the Philippines Los Baños, with the assigned protocol number BIOTECH-2021-001. Additionally, the collection of samples for this study was permitted by each farm owner prior to the sample collection from their pigs. All experiments conducted in this study are in accordance with the relevant regulations, guidelines, and protocols, as evaluated and approved by the UPLB-IACUC.

### Sampling collection

The Philippines is generally divided into three major island groups (Luzon, Visayas, and Mindanao), further subdivided into regions and provinces. While the specific relationships between and among provinces within and among these island groups in relation to trade and transport of live and slaughtered pigs are unknown, it is assumed that a significant percentage of these movements are regional^96^.

Using a multistage stratified random sampling approach, it was determined that a minimum of 648 farms were to be sampled based on Cochran’s method (1977) with the following assumptions: assumed prevalence (P): 0.3, confidence level (1-α): 0.95, margin of error (α): 0.5, and design effect (DE): 2.0^97^.

Based on the Philippine Statistics Authority’s Swine Situation Report as of January 1, 2019^98^, provincial pig population densities were determined, and high hog production provinces were identified. A random sample of three high hog production provinces was selected from each of the three geographical island groups. The provinces randomly selected were Marinduque, Albay, and Batangas for Luzon, Iloilo, Bohol, and Cebu for Visayas, and Misamis Occidental, Zamboanga del Norte, and Misamis Oriental for Mindanao.

Provincial government authorities were requested for a list of villages or barangays that have at least 24 small hold farms. Within these provinces, a random sample of three villages or barangays was selected, and a random sample of 24 households/backyard farms was selected from each barangay. Up to three samples per age group of pigs were collected whenever possible. A sampled pig was classified as either a sow, boar, piglet, or grower by adapting local classification and farming practice. Young pigs housed with the sow and feeding on her milk are considered piglets. A pig housed separately from the sow (i.e., weaned) is considered a grower since distinction among weaners, growers, and finishers is rarely locally applied.

The sampled pigs were manually restrained, and a nose snare and a mouth gag were used to open the mouth and prevent biting. Oral swab samples were collected by rubbing a cotton swab against the oral cavities of the pigs, particularly on the surface of the palatine tonsils whenever safely possible, following the approved protocol identified above. The collection of the swab samples was performed under the guidance of provincial/municipal veterinarians or veterinary technicians. Samples were immediately placed in transport tubes containing 4.0 mL of Amies transport medium. All samples were stored and transported in insulated coolers and immediately processed for streptococcal isolation afterward.

### Isolation and Identification

Serial dilutions of the transport medium were prepared, and 100 µL aliquots of varying dilutions were spread plated onto Columbia Agar plates with 5% defibrinated sheep blood. The plates were then incubated at 37 °C under 5% CO_2_ for 18–24 h. Up to eight α-hemolytic colonies were selected and purified from each sample^25–29,36,37^. All successfully purified isolates were identified using a conventional PCR assay utilizing *S. suis*-specific *recN* gene primers, as described elsewhere^99^.

Partial *16 s rRNA* gene sequences of selected isolates were determined by Sanger sequencing following the amplification of the said gene using the F1/R13 primers, as described elsewhere^100^. Sequences were submitted to the NCBI Genbank database with accession numbers OR287118–OR287128. To better determine sequence similarity, the 16 s rRNA gene sequences of reference isolates of relevant *Streptococcus* species^101^ were also retrieved from the GenBank. Sequences obtained from this study and the retrieved representative sequences from NCBI were aligned using ClustalW method in Molecular Evolutionary Genetics Analysis (MEGA-X) software^102^. Neighbor-joining method^103^ with bootstrap analysis of 1000 replications was used to construct a phylogenetic tree.

### Molecular characterization

Further characterization of the identified *S. suis* isolates was conducted using serotyping, virulence gene profiling, and multilocus sequence typing (MLST). The serotyping of all identified *S. suis* isolates was carried out using four multiplex PCR assays^19^, all targeting capsular polysaccharide synthesis (*cps*) genes located on a single locus in the *S. suis* chromosome. These assays can identify the currently recognized 29 *S. suis* serotypes.

All *S. suis* isolates were similarly profiled for the presence of virulence-associated genes using a multiplex PCR assay that targets the extracellular protein factor (*epf*)*,* muramidase-release protein (*mrp*)*,* and suilysin (*sly*)^104^, commonly referred to as virulence-associated genes (VAGs). The MLST was done using multiplex PCR protocol targeting seven housekeeping genes, including *aroA* (EPSP synthase), *cpn60* (60-kDa chaperonin), *dpr* (peroxide resistance), *gki* (glucose kinase), *mutS* (DNA mismatch repair enzyme), *recA* (homologous recombination), and *thrA* (aspartokinase)^20^. The PCR products were subsequently submitted for sequencing. One hundred twenty-five serotypeable isolates were sequenced for MLST. The remaining six serotypeable isolates did not yield amplicons for one or more of the target genes and thus, were not typed by MLST. An additional 20 nontypeable isolates were selected in random and similarly sequenced. Recovered sequences were submitted to pubMLST (March–May 2023) for verification and typing assignment. The complete dataset pertaining to the isolates, typed by MLST, including provenance/relevant metadata and sequences, can be accessed through the pubMLST database under ID numbers 3539-3546, 3550-3606 and 3660-3739.

The entire *S. suis* database in pubMLST (available on August 13, 2023) was downloaded and used to visualize and identify groups of related genotypes and clonal complexes using the goeBURST algorithm in the PHYLOViZ software^21^.

### Statistical analysis

Data were stored in an in-house Excel 365 (Microsoft) database. Descriptive statistics related to isolation rates per province, farm, and age group as well as the proportion of each serotype among the isolates, were calculated using the same software. Confidence interval estimates of the proportion were calculated with a confidence level of 95%. Meanwhile, in categories with zero observations of the character of concern, the rule of three states that the upper limit of the 95% confidence interval can be estimated by 3/*n*^44,105^.

### Supplementary Information

Supplementary Information.

## Data Availability

The datasets generated in the current study pertaining to the isolates typed by MLST, including provenance/relevant metadata and sequences, can be accessed through the pubMLST database under ID numbers 3539-3546, 3550-3606 and 3660-3739, while the generated and reported partial *16 s rRNA* sequences can be accessed through the NCBI database with GenBank accession numbers OR287118-OR287128. All data presented in this study are included in this article.
