# Supplementary material for: Epidemiology and genetic diversity of Streptococcus suis in smallhold swine farms in the Philippines
Source: Sci Rep. 2023 Dec 1;13:21178. doi: 10.1038/s41598-023-48406-9 (PMC10692119; doi:10.1038/s41598-023-48406-9)
Supplement: Supplementary file 1 — Supplementary Information. [file 41598_2023_48406_MOESM1_ESM.docx]

**Supplemental Table 1.** Frequency counts of sampled farms and pigs and the respective proportion of *S. suis*-positive samples by microbiological isolation per province.

| Province | Sampled Farms  (count) | Positive Farms  (count; %) | Sampled Pigs  (count) | Positive Pigs (count; %) |
| --- | --- | --- | --- | --- |
| Albay | 73 | 6; 8.2% | 153 | 6; 3.9% |
| Batangas | 72 | 16; 22.2% | 194 | 20; 10.3% |
| Bohol | 72 | 17; 23.6% | 176 | 19; 10.8% |
| Cebu | 78 | 7; 9.0% | 259 | 6; 2.3% |
| Iloilo | 73 | 1; 1.4% | 149 | 1; 0.7% |
| Marinduque | 73 | 16; 21.9% | 204 | 20; 9.8% |
| Misamis Occidental | 72 | 5; 6.9% | 159 | 6; 3.8% |
| Misamis Oriental | 73 | 9; 12.3% | 111 | 8; 7.2% |
| Zamboanga del Norte | 78 | 28; 35.9% | 162 | 34; 21.0% |
| Total | 664 | 105; 15.8% | 1567 | 119; 7.6% |


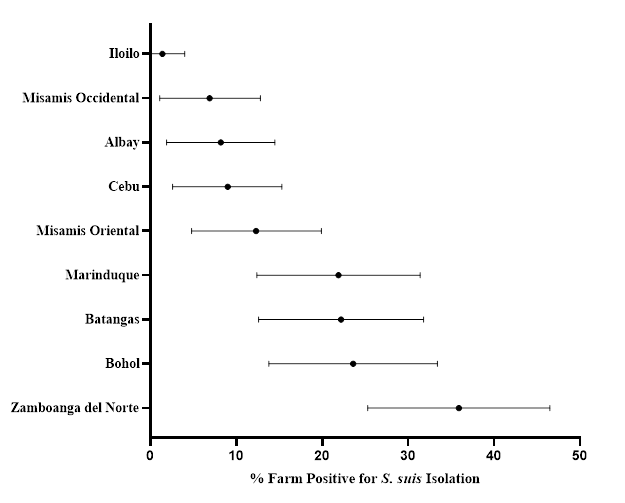


**Supplemental Figure 1.** Confidence interval estimates (95% CI) of the proportion of farm positive for *S. suis* isolation by province.


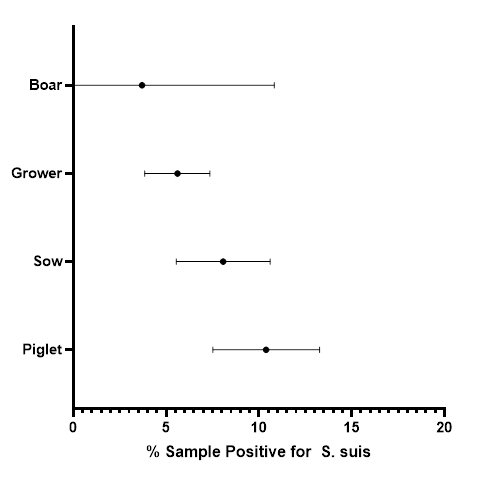


**Supplemental Figure 2.** Confidence interval estimates (95% CI) of the proportion for *S. suis* isolation by age-group.


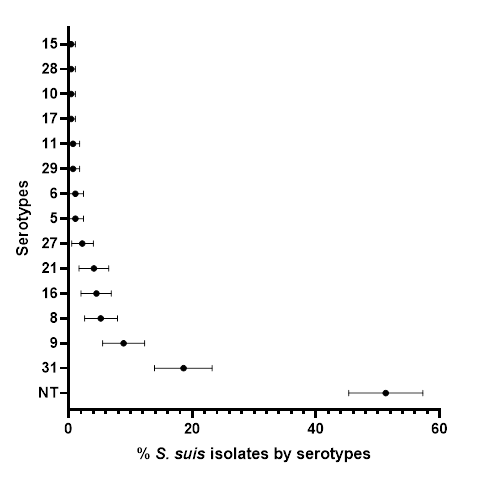


**Supplemental Figure 3.** Confidence interval estimates (95% CI) of the proportion of *S. suis* isolates by serotype.


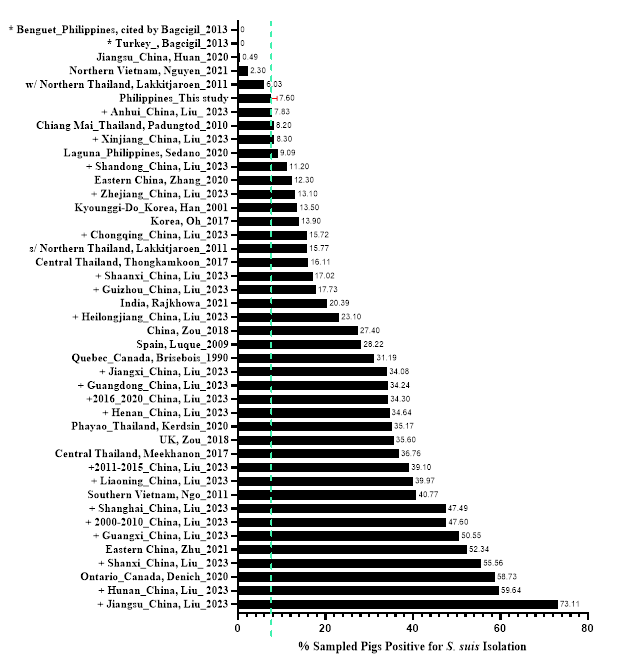


**Supplemental Figure 4.** Reported prevalence of *S. suis* recovery by microbiological isolation from diseased and non-diseased pigs. Indicated by the green line is the estimate of the proportion of pigs positive in the Philippines as observed in this study.

**Notes:**

**+** from meta-analysis pooling all studies in the identified area, clinical status of sample unidentified
s/ - summer time
w/ - winter time

References: ^1–19^


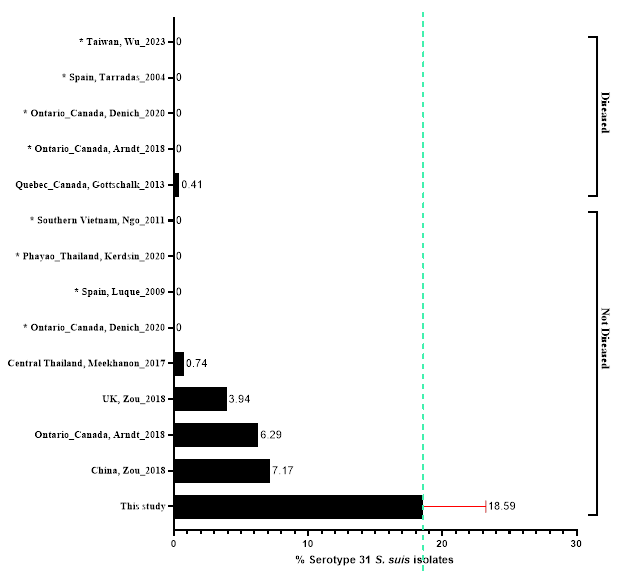


**Supplemental Figure 5a**. Reported proportion of Serotype 31 *S. suis* isolates detected by microbiological isolation from diseased and not diseased pigs. Indicated by the green line is the estimate of the proportion of serotype 31 *S. suis* in the Philippines as observed in this study.

References: ^3,4,6–9,20–23^


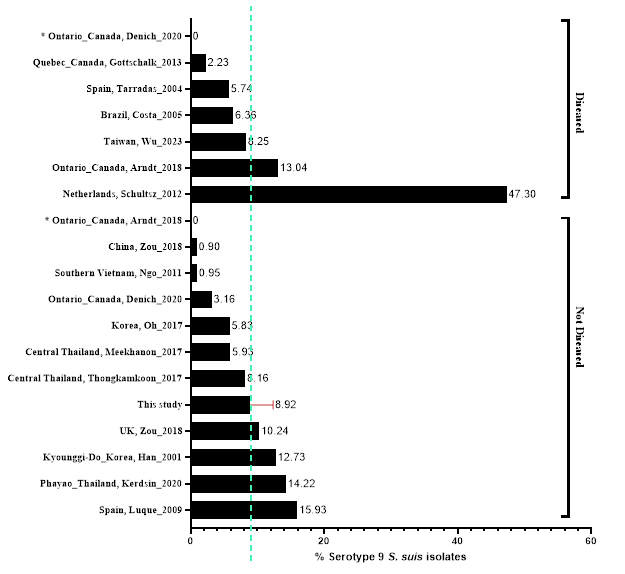


**Supplemental Figure 5b.** Reported proportion of Serotype 9 *S. suis* isolates detected by microbiological isolation from diseased and not diseased pigs. Indicated by the green line is the estimate of the proportion of serotype 9 *S. suis* in the Philippines as observed in this study.

References: ^1,3,21–25,4,6–9,17,18,20^


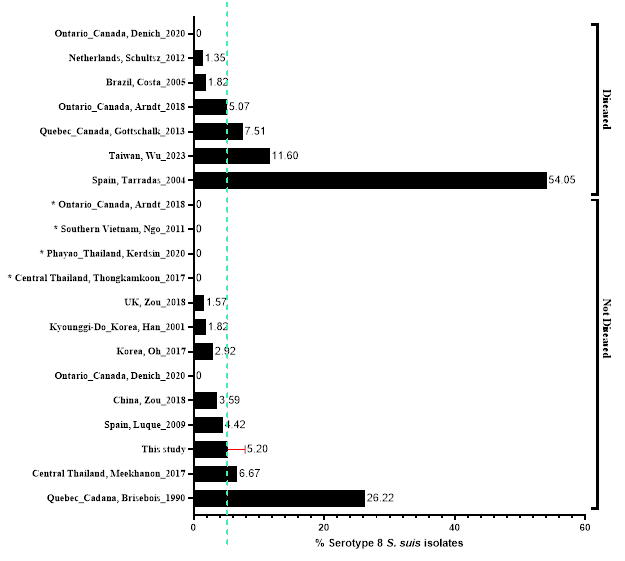


**Supplemental Figure 5c.** Reported proportion of Serotype 8 *S. suis* isolates detected by microbiological isolation from diseased and not diseased pigs. Indicated by the green line is the estimate of the proportion of serotype 8 *S. suis* in the Philippines as observed in this study.

References: ^1,3–9,17,18,20–25^


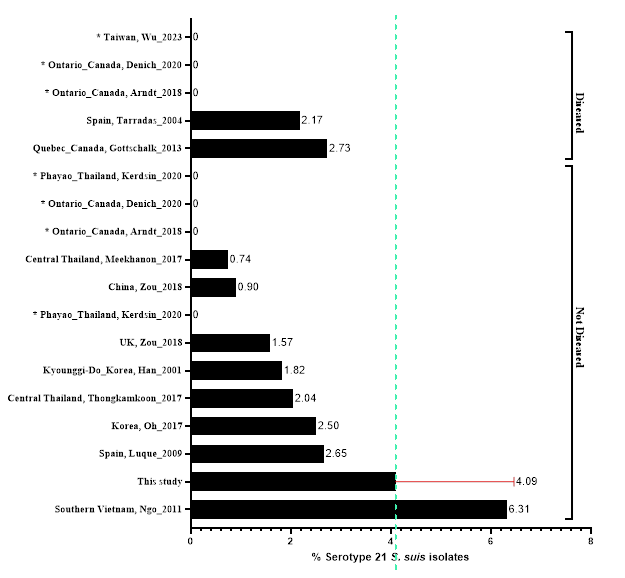


**Supplemental Figure 5d.** Reported proportion of Serotype 21 *S. suis* isolates detected by microbiological isolation from diseased and not diseased pigs. Indicated by the green line is the estimate of the proportion of serotype 21 *S. suis* in the Philippines as observed in this study.

References: ^1,3,21–23,4,6–9,17,18,20^

**
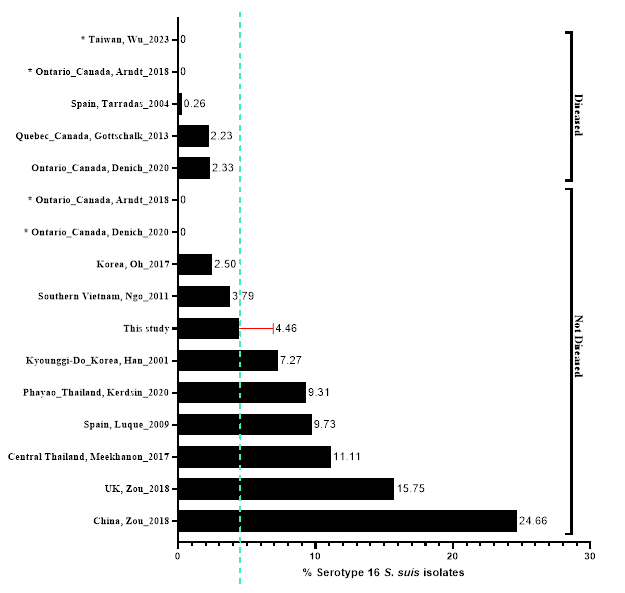
**

**Supplemental Figure 5e**. Reported proportions of Serotype 16 *S. suis* isolates detected by microbiological isolation from diseased and not diseased pigs. Indicated by the green line is the estimate of the proportion of serotype 16 *S. suis* in the Philippines as observed in this study.

References: ^1,3,22,23,4,6–9,17,20,21^

**References**

1. Han, D. U. *et al.* Prevalence, capsular type and antimicrobial susceptibility of Streptococcus suis isolated from slaughter pigs in Korea. *Can. J. Vet. Res.* **65**, 151–155 (2001).

2. Huan, H., Jiang, L., Tang, L., Wang, Y. & Guo, S. Isolation and characterization of Streptococcus suis strains from swine in Jiangsu province, China. *J. Appl. Microbiol.* **128**, 1606–1612 (2020).

3. Zou, G. *et al.* Effects of environmental and management-associated factors on prevalence and diversity of Streptococcus suis in clinically healthy pig herds in China and the United Kingdom. *Appl. Environ. Microbiol.* **84**, 1–15 (2018).

4. Luque, I. *et al.* Genetic analysis of Streptococcus suis isolates recovered from diseased and healthy carrier pigs at different stages of production on a pig farm. *Vet. J.* **186**, 396–398 (2010).

5. Brisebois, L. M., Charlebois, R., Higgins, R. & Nadeau, M. Prevalence of Streptococcus suis in four to eight week old clinically healthy piglets. *Can. J. Vet. Res.* **54**, 174–177 (1990).

6. Kerdsin, A. *et al.* Genotypic comparison between streptococcus suis isolated from pigs and humans in Thailand. *Pathogens* **9**, 4–11 (2020).

7. Meekhanon, N., Kaewmongkol, S., Phimpraphai, W., Okura, M. & Osaki, M. Potentially hazardous Streptococcus suis strains latent in asymptomatic pigs in a major swine production area of Thailand. *J. Med. Microbiol.* **66**, 662–669 (2017).

8. Ngo, T. H. *et al.* Slaughterhouse pigs are a major reservoir of Streptococcus suis serotype 2 capable of causing human infection in Southern Vietnam. *PLoS One* **6**, 1–7 (2011).

9. Denich, L. C. *et al.* A case-control study to investigate the serotypes of s. Suis isolates by multiplex PCR in nursery pigs in Ontario, Canada. *Pathogens* **9**, (2020).

10. Padungtod, P. *et al.* Incidence and presence of virulence factors of streptococcus suis infection in slaughtered pigs from Chiang Mai, Thailand. *Southeast Asian J. Trop. Med. Public Health* **41**, 1454–1461 (2010).

11. Zhu, H. *et al.* Co‐infection analysis of bacterial and viral respiratory pathogens from clinically healthy swine in Eastern China. *Vet. Med. Sci.* **7**, 1815–1819 (2021).

12. Baǧcigil, A. F. *et al.* Isolation of streptococcus species from the tonsils of slaughtered pigs. *Turkish J. Vet. Anim. Sci.* **37**, 94–96 (2013).

13. Nguyen, N. T. T. *et al.* An epidemiological study of Streptococcus suis prevalence among swine at industrial swine farms in Northern Vietnam. *One Heal.* **13**, 1–5 (2021).

14. Lakkitjaroen, N. *et al.* Prevalence and antimicrobial susceptibility of Streptococcus suis isolated from slaughter pigs in Northern Thailand. *Kasetsart J. - Nat. Sci.* **45**, 78–83 (2011).

15. Liu, P., Zhang, Y., Tang, H., Wang, Y. & Sun, X. Prevalence of Streptococcus suis in pigs in China during 2000–2021: A systematic review and meta-analysis. *One Heal.* **16**, (2023).

16. Zhang, C. *et al.* Capsular serotypes, antimicrobial susceptibility, and the presence of transferable oxazolidinone resistance genes in Streptococcus suis isolated from healthy pigs in China. *Vet. Microbiol.* **247**, (2020).

17. Oh, S. I. *et al.* Capsular serotypes, virulence-associated genes and antimicrobial susceptibility of Streptococcus suis isolates from pigs in Korea. *J. Vet. Med. Sci.* **79**, 780–787 (2017).

18. Thongkamkoon, P., Kiatyingangsulee, T. & Gottschalk, M. Serotypes of Streptococcus suis isolated from healthy pigs in Phayao Province, Thailand. *BMC Res. Notes* **10**, 1–7 (2017).

19. Rajkhowa, S. & Rajesh, J. B. Virulence associated gene profiling and antimicrobial resistance pattern of Streptococcus suis isolated from clinically healthy pigs from North East India. *Lett. Appl. Microbiol.* **73**, 392–397 (2021).

20. Gottschalk, M. *et al.* Characterization of Streptococcus suis isolates recovered between 2008 and 2011 from diseased pigs in Québec, Canada. *Vet. Microbiol.* **162**, 819–825 (2013).

21. Wu, C. F. *et al.* Serotype and multilocus sequence typing of Streptococcus suis from diseased pigs in Taiwan. *Sci. Rep.* **13**, 8263 (2023).

22. Tarradas, C. *et al.* Distribution of serotypes of Streptococcus suis isolated from diseased pigs in Spain. *Vet. Rec.* **154**, 665–666 (2004).

23. Arndt, E., Farzan, A., Slavićc, D., Maclnnes, J. & Friendship, R. An epidemiological study of Streptococcus suis serotypes of pigs in Ontario determined by a multiplex polymerase chain reaction. *Can Vet J* **59**, 997–1000 (2018).

24. Schultsz, C. *et al.* Differences in the population structure of invasive streptococcus suis strains isolated from pigs and from humans in the Netherlands. *PLoS One* **7**, 5–10 (2012).

25. Costa, A. T. R. *et al.* Serotyping and evaluation of the virulence in mice of Streptococcus suis strains isolated from diseased pigs. *Rev. Inst. Med. Trop. Sao Paulo* **47**, 113–115 (2005).
